# Alpha-1 antitrypsin deficient individuals have circulating extracellular vesicles with profibrogenic cargo

**DOI:** 10.1186/s12964-020-00648-0

**Published:** 2020-09-04

**Authors:** Nazli Khodayari, Regina Oshins, L. Shannon Holliday, Virginia Clark, Qiang Xiao, George Marek, Borna Mehrad, Mark Brantly

**Affiliations:** 1 Division of Pulmonary, Critical Care, and Sleep Medicine, University of Florida, Gainesville, USA; 2 College of Dentistry, University of Florida, Gainesville, USA; 3 Division of Gastroenterology, Hepatology, and Nutrition, University of Florida, Gainesville, USA; 4 MilliporeSigma, Burlington, MO USA

**Keywords:** Alpha-1 antitrypsin, Extracellular vesicles, Cytokine, miRNA, Liver fibrosis

## Abstract

**Background:**

Alpha-1 antitrypsin deficiency (AATD)-mediated liver disease is a toxic “gain-of-function” inflammation in the liver associated with intracellular retention of mutant alpha-1 antitrypsin. The clinical presentation of the disease includes fibrosis, cirrhosis and liver failure. However, the pathogenic mechanism of AATD-mediated liver disease is not well understood. Here, we investigated the role of plasma extracellular vesicles (EVs) in progression of AATD-mediated liver disease.

**Methods:**

EVs were isolated from plasma of AATD individuals with liver disease and healthy controls. Their cytokines and miRNA content were examined by multiplex assay and small RNA sequencing. The bioactivity of EVs was assessed by qPCR, western blot analysis and immunofluorescent experiments using human hepatic stellate cells (HSCs) treated with EVs isolated from control or AATD plasma samples.

**Results:**

We have found that AATD individuals have a distinct population of EVs with pathological cytokine and miRNA contents. When HSCs were cultured with AATD plasma derived-EVs, the expression of genes related to the development of fibrosis were significantly amplified compared to those treated with healthy control plasma EVs.

**Conclusion:**

AATD individuals have a distinct population of EVs with abnormal cytokine and miRNA contents and the capacity to activate HSCs and mediate fibrosis. Better understanding of the components which cause liver inflammation and fibrogenesis, leading to further liver injury, has the potential to lead to the development of new treatments or preventive strategies to prevent AATD-mediated liver disease.

**Video abstract**

## Background

Alpha-1 antitrypsin (AAT), is primarily produced in the liver, contributing to the innate immune system as an anti-inflammatory protein by inhibiting destructive neutrophil proteases [1]. Moreover, AAT has been shown to have anti-inflammatory properties independent of its antiprotease activity through modulating cellular processes like apoptosis and cytokine expression [2, 3]. Alpha-1 antitrypsin deficiency (AATD) is a common inherited cause of liver disease and the most frequent genetic etiology for pediatric liver disease and transplantation [3]. Z mutation - the most common deficiency allele - results in accumulation of misfolded ZAAT in hepatocytes and monocytes and low levels of circulating AAT which, leads to uncontrolled proteolytic activity in different tissues especially in lungs [1]. The histological features of the liver disease are characterized by the accumulation of aggregated ZAAT within hepatocytes accompanied by hepatic injury and inflammation. A “two-hit model” has been postulated as a potential mechanism responsible for liver disease pathogenesis, in which the first “hit” is provided by accumulation of misfolded ZAAT in the endoplasmic reticulum (ER) of hepatocytes, and the second hit, which can include oxidative stress and inflammation [4]. The extrahepatic inflammation may affect liver function via secreted soluble factors leading to the activation of inflammatory pathways and facilitating progression of hepatic injury [5]. Studies from human AAT transgenic mice show that intrahepatic accumulation of ZAAT is associated with liver injury in these mice [6]. We have also shown a complex relationship between the accumulation of ZAAT, steatosis, and fibrosis in AATD individuals [7].

Hepatic stellate cells (HSCs) localize to the perisinusoidal space and are the primary source of activated myofibroblasts that drive liver fibrogenesis. Activated HSCs are a major source of collagen, ECM proteins, and tissue inhibitors of metalloproteinases promoting liver remodeling in response to inflammatory mediators released from neighboring cells [8] as well as cells from different organs [9]. Although the mechanisms defining fibrogenesis initiated by HSCs is unclear, recent studies have explored potential roles for Extracellular Vesicles (EVs) in the pathogenesis of liver fibrosis initiated by activation of HSCs [10].

EVs are membrane vesicles naturally released from every cell type, with diameters ranging from 30 to 200 nm. They are enriched in nucleotides, lipids, and proteins from their cells of origin which characterizes them as distinct organelles [11]. They act as local immune modulators by delivering their cargo to adjacent recipient cells or enter in a biological fluid thus reaching distant sites [11]. Recent research has implicated that EVs released by activated immune cells and injured parenchymal cells act in a paracrine manner [12] to stimulate liver fibrosis. Signs of immune-mediated complications are uniquely present in AATD patients [13, 14] and suggest a contribution from a variety of activated immune cells as well as liver parenchymal cells. Thus, whether EVs may be modulated by AATD disease state remains to be determined as does the contribution of circulating modulated EVs to immune responses in AATD-mediated liver disease.

In this study we test the hypothesis that immune complications and injured hepatocytes associated with AATD, results in release of EV population carrying pathological cargo. We show that the AATD individuals exhibit a pro-fibrogenic EV cytokine and miRNA profile. Furthermore, we show that AATD plasma derived EVs have the ability to promote liver fibrosis by inducing HSCs migration, trans-differentiation and activation in vitro. Our results suggest that in the pathogenesis of AATD-mediated liver disease, EVs play an important role in modulating a cross-talk network in liver fibrosis. These findings begin to explain how immune complications associated with AATD may regulate organ cross-talk and provide a mechanism by which circulating EVs carrying pathological cargo modulates HSCs activation initiating liver fibrosis.

## Material and methods

### Clinical samples

Age and sex matched healthy subjects with an MM genotype and no family history of AATD were selected from the Alpha-1 Foundation DNA and Tissue Bank for use as a control group. Study subjects were chosen from a clinical study based at the University of Florida that followed patients with AATD over a 3-year period to evaluate progression of liver disease (Table 1). Plasma from subjects with a ZZ genotype and liver biopsies indicating presence of fibrosis and high levels of PASD positive cells were used [7]. All AATD individuals confirmed by genetic testing who were age 21–71, from the US or Canada, and willing to consent to a liver biopsy were eligible. Decompensated liver disease (ascites, variceal bleeding, hepatic encephalopathy, hepatocellular carcinoma) was excluded. Recipients of a liver or lung transplant were excluded. A total of 26 patients were included. All participants gave informed, written consent prior to enrolling. The study was approved by the University of Florida institutional IRB [13]. All authors had access to the study data and reviewed and approved the final manuscript.

**Table 1:** Clinical features of study subjects

Variables	Controls *n* = 20	Patients *n* = 20
Age	47.0 ± 14.2	60.4 ± 7.7
Gender		
Male (%)	6 (30)	14 (70)
Female (%)	14 (70)	6 (30)
Smoking History		
Never (%)	4 (20)	12 (60)
Current (%)	3 (15)	0 (0)
Past (%)	4 (20)	8 (40)
No data (%)	9 (45)	0 (0)
Genotype	MM = 20 (100)	ZZ = 20 (100)
PASD 3(%)	No biopsy	20 (100)
Fibrosis		
3	No biopsy	6 (30)
2		10 (50)
1		4 (20)
Plasma AAT Levels (μM)	24.7 ± 4.9	13.8 ± 10.9

Data are expressed as mean (SD) or n (percentage)

### Cell culture

Human LX-2 cell line, purchased from MilliporeSigma (Burlington, MA) were cultured in Dulbecco’s Modified Eagle’s Medium (DMEM) supplemented with 2% FBS and 1% Primocin™ from InvivoGen (San Diego, CA), at 37 °C in a humidified incubator under 5% CO2. iPSC-derived HSCs were differentiated from induced pluripotent stem cells (iPSCs) [15]. Briefly, peripheral blood mononuclear cells-derived iPSCs provided by iPSC core at the University of Florida were incubated with BMP4 from StemCell Technologies (Vancouver, BC) for 4 days to induce mesodermal progenitors. Following this step, the cells were subsequently incubated with BMP4, FGF1, and FGF3 until day 6 to induce a liver mesenchymal submesothelial phenotype. From days 6 to 8, the cells were incubated with FGF1, FGF3, retinol, and palmitic acid, from StemCell Technologies (Vancouver, BC). The final step of differentiation was modeled by incubation of the cells with retinol and palmitic acid from day 8 to day 12. We next characterized differentiated iPSC-HSCs on day 12 (Supplementary Figure 1a and B). iPSC-HSCs were analyzed for the expression levels of markers associated with HSCs (Supplementary Figure 1C) including *TIMP (Tissue Inhibitor of Metallopeptidase Inhibitor 1), CoL1A1(Collagen type1 Alpha1), α*-*SMA (Alpha Smooth Muscle Actin) and LRAT (Lecithin: Retinol Acyl Transferase).*

### Isolation of EVs

EVs were isolated from approximately 3 mL of cryopreserved plasma by differential centrifugation [16]. Briefly, the plasma was diluted in PBS (1:1) and centrifuged at 2000 x g for 30 min at 4 °C and at 12,000 rpm for 30 min at 4 °C. Supernatants were collected into ultracentrifuge tubes and centrifuged in a Beckman SW-40Ti swinging rotor ultracentrifuge (Beckman Coulter) at 110,000 x g for 2 h at 4 °C. Resuspended pellets in PBS were filtered through a 0.22-μm filter (Millipore, Billerica, MA) and centrifuged at 110,000×g for 70 min at 4 °C. EV pellets were washed with PBS and centrifuged at 110,000×g for 70 min at 4 °C and resuspended in 200 μL of PBS. EV preparations were conserved at − 80 °C for later use.

### Particle number and size measurement

The size distribution and concentration of the isolated EVs was analyzed by Nanosight NS300 system (Nanosight and Malvern, United Kingdom). Briefly, purified EVs were homogenized by vortexing followed by dilution of 1:100 in sterile PBS. Each sample analysis was conducted for 60 s. Data was analyzed by Nanosight NTA 2.3 Analytical Software with the detection threshold optimized for each sample and screen gain at 10 to track as many particles as possible with minimal background. A blank 0.2 μm- filtered 1x PBS was also run as a negative control. At least five analysis were done for each individual sample.

### Transmission electron microscopy

Purified EVs were fixed with 2% paraformaldehyde. A 20 μl drop of the suspension was loaded onto a formvar coated grid, negatively stained with 2% aqueous uranyl acetate, and examined under a Hitachi 7600 transmission electron microscope (Hitachi High-Technologies, Schaumburg, IL) equipped with a Macrofire monochrome progressive scan CCD camera (Optronics, Goleta, CA) and AMTV image capture software (Advanced Microscopy Techniques, Danvers, MA).

### EVs RNA isolation, small RNA library preparation, sequencing, and bioinformatics

RNA was isolated using exoRNeasy Maxi kit (Qiagen) from purified EV fractions derived from 4 MM healthy and 5 ZZ plasma samples. The quantity and quality of the RNA were determined using the Agilent RNA 6000 Pico Kit and a Small RNA Kit Chip on the Agilent Bioanalyzer instrument (Agilent Technologies). Sequencing libraries were constructed using ∼2 ng of total EV RNA with Small RNA Library Prep Set for Illumina kit (New England BioLabs) and were sequenced using Illumina Miseq 1 × 150 cycles V3 kit at the University of Florida Interdisciplinary Center for Biotechnology Research. The expression of the miRNAs was obtained using miRDeep2 software [14] and differential analysis was performed using the exact test from edgeR package [17]. We performed enrichment analysis using Ingenuity Pathway Analysis (IPA) (Ingenuity Pathways Analysis (IPA) system. Redwood, CA: Ingenuity Systems, Inc.;) package using the miRNA-mRNA target link module to link the discovered significantly differentially expressed miRNAs with their mRNA target genes. Because IPA does not include prediction scores, to validate the miRNA target genes, we performed additional computational analysis using TargetScan (Release 7.1. Jun, 2016.) to identify target genes with good predicted scores. In addition, we used IPA to identify miRNA-mRNA target genes which have been experimentally confirmed.

### Real-time qPCR validation of selected miRNAS from small RNA-sequencing

Reverse transcription reactions were performed using the TaqMan Advanced miRNA Assays kit (Applied Biosystems. Inc., CA; USA) using 2 μL of cell-free RNA extracted from exosomes. Real-time PCR reactions were performed in duplicate in 20 μL reaction volumes using 10 μL SensiFAST Probe Lo-ROX 2x PCR Master Mix (Bioline, Memphis, TN), 1 μL TaqMan Advanced miRNA assay (20x) (Applied Biosystems. Inc., CA; USA), 4 μL of nuclease free water and 5 μL of RT product after a 1:10 dilution. Real-time PCR was carried out on an Applied BioSystems 7500 Fast thermocycler (Applied Biosystems. Inc., CA; USA) programmed as follows: 95 °C for 20 s followed by 40 cycles of 95 °C for 3 s and 60 °C for 30 s. We used hsa-miR-21-5p and hsa-miR-26a-5p, two of the most stable miRNAs in terms of read counts, as a endogenous controls. All fold-change data were obtained using the delta-delta CT method (2 − ΔΔCT) 28. Five differentially expressed miRNAS detected after small RNA-seq were validated using RT-qPCR (hsa-miR-128-3p, hsa-miR-6809-5p, hsa-miR-125a-5p, hsa-miR-151a-5p and hsa-miR-99a-5p).

### Cytokine measurement

We first identified priority cytokine candidates based on known liver fibrosis pathophysiology and previously published literature, then selected from the assays available at Myriad-RBM, which were primarily multiplexes (Luminex, Myriad-RBM Inc., Austin TX). Then purified EVs from 20 MM and 20 ZZ plasma samples (Table 1), were suspended in a mixture of water and PBS (1:2). EVs in solution were lysed using an equal volume of IP lysis buffer. Lysed EV samples to which Triton X was added at final concentration of 1% were run on the MilliPLEX Human High Sensitivity T Cell Magnetic Bead Panel Luminex kit for measurement of 21 unique cytokines per plex according to manufacturer’s instructions (EMD Millipore, St. Louis, MO).

### Labeling of EVs and uptake

Purified EVs from plasma (10^9^ CD63-positive particles) were incubated with Fast DiO membrane dye (Invitrogen) at a final concentration of 2 μg/mL for 1 h at room temperature. The purification process of washing and ultracentrifugation was repeated twice before the labeled EV pellet was resuspended in PBS [18]. For microscopic analysis, LX2 cells were incubated with DiO-labeled EVs for 1 h at 37 °C. After incubation, cells were washed with PBS to remove unbound labeled EVs and subsequently imaged with a Keyence fully motorized BZ-X800 microscope (KEYENCE America, Chicago, USA).

For flow cytometry analysis, LX2 cells were seeded in six-well plates and grown overnight and DiO-labeled EVs (2 × 10^7^) in 100 μL PBS/well (CD63-positive) were added and incubated at 37 °C for 1 h. Cells stained directly with 1 μL/well DiO (1 μg/mL) served as a positive control, and unstained cells as a negative control. Cells were analyzed using a Beckman Coulter Gallios Flow Cytometer using Kaluza acquisition software and Flow Jo for analysis. Each experimental group was performed in triplicate.

### Co-incubation of human plasma-derived EVs with hepatic stellate cells

HSCs were plated at the cell concentration of 200,000 per well in 12-well plates. Isolated EVs (pooled from 5 control or AATD individuals (isolated from 250 μL plasma) were added to each well and the plate was incubated for 48 h at 37 °C [16]. HSCs were incubated with the same volume of PBS and have been used as negative control.

### Cell migration and proliferation assay

Cell migration was assessed by measuring the repair of a linear wound generated in the confluent monolayer of cells. Hepatic stellate cells were (LX-2) plated at an equivalent density on chamber slides (Lab-Tek, Westmont, IL), and incubated with or without plasma derived EVs for 4 h before the application of a linear scratch in cell monolayers using a sterile plastic pipette tip. Cell migration was recorded for 4, 8, 16 and 24 h using an Applied Precision deconvolution microscope. To assess the proliferation rate of the cells, 4 different fields of each condition has been subjected to cell counting for 4, 8, 16 and 24 h and the mean values have been recorded as the total number of cells.

### Quantitative real-time PCR

Total RNA was extracted using Trizol reagent (Takara, A7603–1), and reversely transcribed through SuperScript VILO™ kit (Invitrogen, Carlsbad, CA). Real-time PCR analyses were performed with SensiFAST qPCR Master Mix (Bioline, Memphis, TN) on a 7500 Real-time PCR system, Applied Biosystems. Probes used for human Acta2 (Hs00426835_g1), Col1a1 (Hs00164004_m1), Timp1 (Hs01092512_g1) and LRAT (Hs00428109_m1) detection were purchased from Thermofisher (Carlsbad, CA).

### Immunoblotting

LX-2 cells and iPSc-derived HSCs were seeded at 3 × 10^5^/well in 6-well plates with or without MM or ZZ plasma-derived EVs for 24 h. Protein levels in the cell lysate homogenates utilizing RIPA buffer were determined using the bicinchoninic acid method (Pierce Biotechnology, Rockford, IL). Rabbit polyclonal antibodies were used to detect total and phospho-IKK [19], p65, p50 [20], total and phospho-STAT [21], total and phospho-JAK [21] and GAPDH (Cell Signaling, Danvers, MA), CD81 [22], CD63 [23] and TSG101 [24] (Proteintech, Chicago, IL). Proteins were detected by using a Super Signal West Dura Extended Duration Substrate Kit from Thermo Scientific.

### Immunostaining and immunofluorescence microscopy

LX-2 cell line treated with or without MM or ZZ plasma-derived EVs were grown on glass coverslips. After 24 h, the cells were fixed with 4% paraformaldehyde in PBS for 20 min. The cells were incubated for 1 h with blocking buffer at room temperature, followed by incubating overnight at 4 °C with primary antibodies (1:400). The cells were washed with 1X PBS and incubated for 1 h with secondary antibodies (Alexa Fluor 488 goat anti-mouse IgG and Alexa Fluor 594 goat anti-rabbit IgG). Images were collected using a Keyence fluorescence microscope (Osaka, Japan). Images were processed for brightness and contrast and filtered for noise following good practices as outlined by Rossner and Yamada.

### Histology, histochemistry and immunohistochemistry

Paraffin-embedded liver tissue sections were routinely de-paraffinized with xylene and a graded series of ethanol. Some tissue sections were stained with hematoxylin–eosin (HE) for simple morphological examination. For immunostaining on paraffin-embedded liver tissues, paraffin blocks were sliced into 5 μm sections, deparaffinized with xylene, and rehydrated with decreasing concentrations of ethanol in water. Antigen retrieval was achieved by incubation for 20 min in hot (95 °C) sodium citrate buffer (pH 6.0) and 20 min of cooling at room temperature. Endogenous peroxidases were quenched by incubation in 3% hydrogen peroxide for 20 min. Sections were washed with PBS. Primary antibodies were applied for 60 min at room temperature in a humidified chamber. After rinsing the slides in PBS, they were incubated in secondary antibody for 1 h at room temperature. After washing with PBS, slides were incubated with Vectastain ABC (Vector Laboratories) for 30 min. After washing with PBS, color development was achieved by applying diaminobenzidine tetrahydrochloride (DAB) (Vector Laboratories) for two to 5 min. Images were acquired and the signal intensity was quantified using a Keyence BZ-X700 microscope.

### Plasma levels of CRP quantification

Plasma samples were centrifuged at 20,000 x g for 10 min to remove fibrin or debris. Samples (100 μL) were then loaded undiluted and CRP levels measured using the Cardio Phase hsCRP assay on a Siemens Nephelometer calibrated with the Rheumatology Standard according to manufacturer’s directions. The assay has a range of 0.1–50 mg/L.

### Plasma levels of CXCL10 quantification

CXCL10 levels were measured in the plasma of ZZ patients with liver disease and MM controls using an ELISA kit from R&D Systems according to manufacturer’s instruction. Samples were diluted 1:2 in assay diluent before being applied to the assay plate for 2 h incubation at room temperature. The plate was washed, Human IP-10 Conjugate added to each well, and the plate incubated for another 2 h at room temperature. The plate was washed again and substrate solution added to each well. The plate was incubated for 30 min in the dark before adding stop solution. The optical density was measured using a SpectraMax spectrometer at 450 nm and the sample concentrations calculated based on the standard curve.

### Liver histology

A percutaneous liver biopsy was performed with a 16 gauge BioPince™ core biopsy needle after using ultrasonography to mark the location. The sample was fixed in formalin and processed for examination. Stains included H&E, trichrome, PAS/PAS + D and Prussian blue. Portal inflammation and hepatocyte degeneration were noted [7].

### FibroScan and ultrasound

Liver stiffness measurements (LSM) were performed by using FibroScan device powered by vibration controlled transient elastography (Echosens). Two individuals performed the procedure after training on proper technique. LSM were considered reliable if the following three criteria were met: at least 10 valid measurements, ≥60% success rate, IQR/median ≤ 30% [13].

### Data analysis

We extracted data on sample size, mean cytokine concentration, standard deviation (SD), and *p*-value to calculate the effective size (ES). The sample size, mean cytokine concentration, and SD were used to calculate the ES. Two-sample t test for mean difference with unequal variance was used to test the power of each group sample size of 20 using the usual significance level alpha = 0.05. Power analysis and statistical analyses were performed using SAS and GraphPadPrism 8 software (San Diego, CA). If cytokine concentration data were not available, the ESs were generated by sample size and *p*-value. All results are expressed as mean ± S.E. Statistical analyses were performed using Prism 8 by Student t-test or Mann-Whitney U test. Values of *P* < 0.05 was considered statistically significant. Power analysis showed the power of > 0.842.

## Results

### Isolation and characterization of plasma EVs

**Fig. 1:**
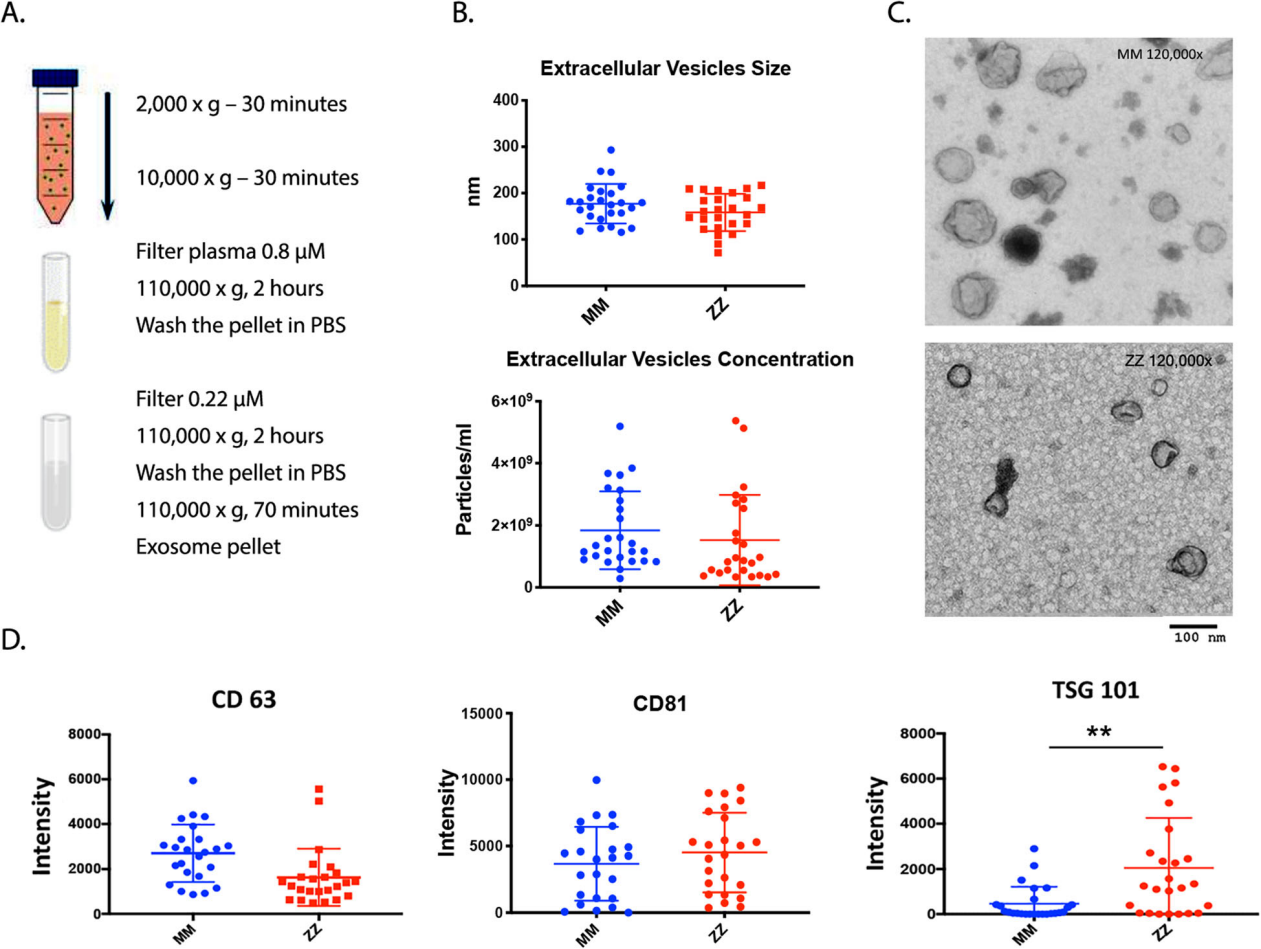
Isolation and characterization of plasma EVs. **a** Ultra-centrifugation was used to isolate EVs from the plasma of normal (MM) and AATD (ZZ) individuals; **b** NTA was performed on plasma EVs purified by ultracentrifugation to determine their concentration (upper panel) or size (nm) (lower panel) in individual groups of MM and ZZ (26 individuals per group); **c** EVs were characterized by TEM; **d** Western blot analysis of EVs using anti-CD 63 (left), anti-CD81 (middle), and anti-TSG 101 antibodies (right). Graphs show quantification of immunoreactive signals by scanning, normalized to the number of the EVs, *p = 0.0013

EVs were isolated from plasma of 26 control subjects (MM) and 26 AATD individuals (ZZ) with liver disease by a combination of filtration and ultracentrifugation (Fig. 1a) and were characterized by analyses for particle size, distribution, and concentration using Nanoparticle tracking analysis (NTA) and transmission electron microscopy (TEM). EVs isolated from both MM and ZZ groups of subjects were within the normal range for EV size (30–200 nm in diameter) and the EV recovery from plasma in ZZ individuals was the same as those in MM control group (Fig. 1b). Negative staining of classic TEM demonstrated cup-shaped, round particles (Fig. 1c). The presence of EV markers was also investigated by western blot analysis. The results confirmed abundant CD63, CD81, and TSG101 expression in all EV fractions. Among detected EV protein markers, TSG101 was enriched in ZZ plasma derived-EVs compared to EVs isolated from MM healthy controls (Fig. 1d).

### Comparison of EV-associated and plasma cytokines and chemokines between control and AATD subjects

**Fig. 2:**
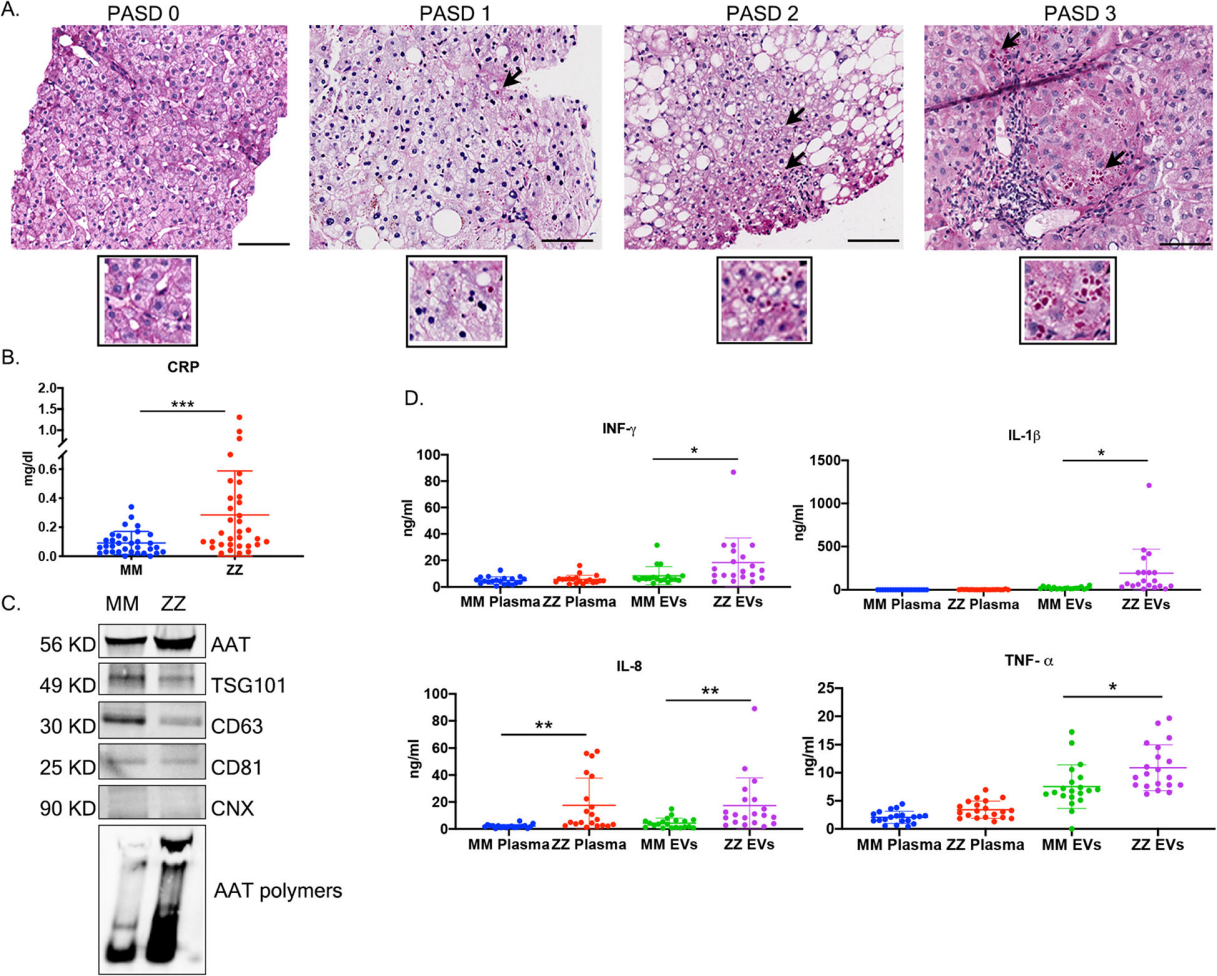
Comparison of EV-associated and plasma cytokines and chemokines between control and AATD subjects. **a** Representative liver biopsies show increasing PAS + D globule grade, indicating ZAAT accumulation. Black arrows highlight cells with PAS-D globules. Scale bar represents 100 μ m. **b** The distribution of CRP levels in MM and ZZ individuals. **c** The levels of total and polymeric AAT in plasma derived EVs pooled from 4 MM and 4 ZZ individuals. Calnexin (CNX) has been loaded as negative marker for EVs. **d** The levels of differential expressed free and EV-associated cytokines in plasma samples from MM individuals, compared to ZZ individuals, *n* = 20, *p = 0.0101, **p = 0.0015, ***p = 0.0003

The diagnosis of AATD individuals was confirmed by phenotyping and the prevalence of clinically significant liver disease was determined by liver biopsy. Histologically, insoluble ZAAT proteins are characterized by globules which stain bright pink with periodic acid–Schiff’s reagent, which are resistant to diastase treatment (PAS + D) (Fig. 2a). PAS + D globules present in a high-power field were scored from 0 to 3 as follows: 0-None, 1-Rare: < 5 hepatocytes with globules, 2-Few: 5–20 hepatocytes with globules, 3-Numerous: ≥20 hepatocytes with globules [7]. IHC staining showed evidence of aggregated ZAAT in all AATD subjects. After evaluating PAS + D accumulation in AATD individuals, we measured CRP levels of healthy control and AATD individuals. The univariate analysis revealed that the AATD individuals are divided into normal (< 0.4 mg/dL) and elevated (≥0.4 mg/dL) CRP levels. Approximately one-third of patients (32%) had CRP levels ≥0.4 mg/dL, indicative of inflammation. All healthy controls (100%) had CRP levels (< 0.4 mg/dL), indicative of a healthy condition with no sign of inflammation (Fig. 2b). Then, utilizing western blot analysis we evaluated the presence of AAT protein in the plasma derived EVs pooled from 4 MM and four ZZ individuals with PAS + D scores of 3 and found that the total levels of AAT is higher in EVs isolated from ZZ individuals. Furthermore, non-denaturing western blot analysis showed that ZZ plasma derived EVs carry significant amounts of AAT aggregates (Fig. 2c). We also evaluated absolute amounts of each cytokine in the EV fraction versus the un-encapsulated (soluble) form from 20 MM and 20 ZZ plasma samples. Distribution of each cytokine between the free and EV-encapsulated form is a characteristic of a system, rather than of the cytokines being secreted by the same pathway in all systems [25]. We found that in our study groups, the levels of EV-associated INF- *γ*, IL1- *β*, and TNF- *α* cytokines were significantly higher in AATD individuals, whereas there was no difference between the levels of free form of the same cytokines in the control group. The level of both free form and EV-associated IL-8 was significantly higher in AATD individuals (Fig. 2d).

### Differential miRNA expression profiles among plasma derived EV miRNAs from healthy and AATD individuals

**Fig. 3:**
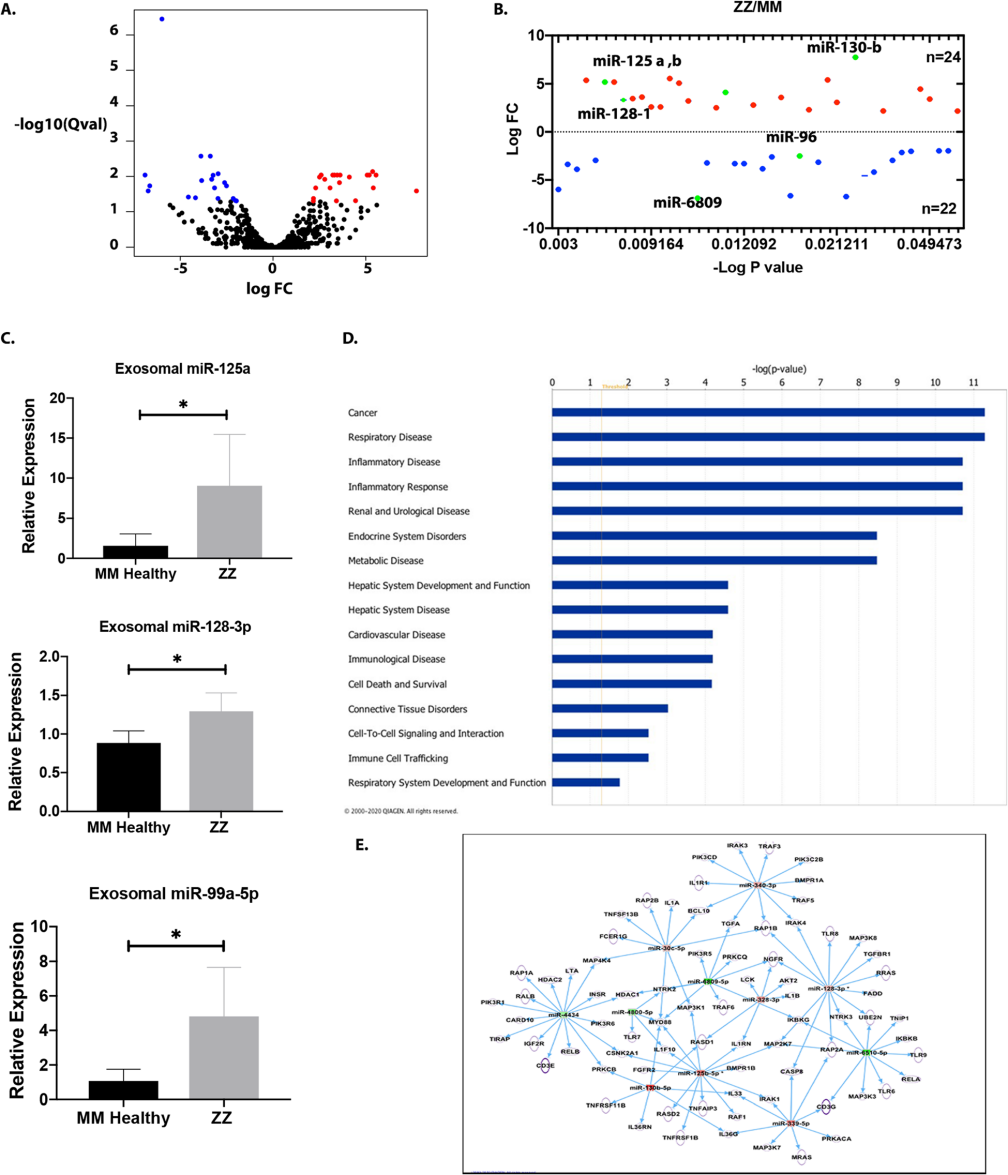
Differential miRNA Expression Profiles among plasma derived EV miRNAs from healthy and AATD individuals with liver disease. **a** Volcano plot of total and **b** differentially expressed miRNAs in ZZ individuals compared with MM healthy controls. Plotted along the x-axis is the mean of log2 fold-change and the y-axis is the negative logarithm of the -log *P*-values. Red points represent significantly upregulated miRNAs and blue points represent significantly downregulated miRNAs. **c** Relative expression of selected miRNAs in the plasma exosomal fraction from MM and ZZ subjects using q-PCR. **d** Bar graph indicating the number of miRNAs that are predicted to target genes in each category listed on the *x*-axis. **d and e** Molecular networks linking highly differentially expressed microRNAs (miRNAs) and their target genes involved in hepatic stellate cell activation between AATD individuals and healthy controls. Upregulated miRNAs are in red and downregulated miRNAs are green

To explore the expression profile of miRNAs in plasma-derived EVs from MM and ZZ individuals, we isolated EVs as previously described. The EVs were then validated by NTA and electron microscopy analyses. We then performed high-throughput sequencing of small-RNA transcriptomes (≤50 nt) for miRNA expression profiles. A total of 869 miRNAs in MM and ZZ groups were identified at the threshold of > 1 CPM (Counts Per Million) in at least half of the samples and the whole distribution of differentially expressed miRNAs was visualized in the volcano plots (Fig. 3a). We identified 44 differentially expressed miRNAs, independent of age and sex, at thresholds of fold change > 2 and q value < 0.05 in the plasma derived EVs from the healthy MM individuals compared with ZZ individuals. Among the differentially expressed miRNAs, 23 were upregulated and 21 were downregulated (Fig. 3b) in the EVs of the ZZ individuals. The top 10 differentially expressed miRNAs were the hsa-miR-125 family, hsa-miR-335-3p, hsa-miR-339-5p, hsa-miR-4433b-5p, hsa-miR-130b-5p, hsa-miR-658, hsa-miR-6809, and hsa-miR-6510-5p (Table 2). Five differentially expressed miRNAs identified by NGS were validated using q-PCR (Fig. 3c). As compared to healthy MM subjects, miR-128-3p, miR-125a-5p and miR-99a-5p were significantly up-regulated in ZZ subjects. These results are congruent to the pattern obtained from miRNA sequencing. To examine the potential biological functions of the select miRNAs, the data was subjected to Ingenuity Pathway Analysis (IPA). The selected genes were then further grouped into functional categories and plotted as a bar graph (Fig. 3d). This analysis revealed several different functional categories including cancer, respiratory disease, immunological disease, inflammatory response, hepatic system disease, and others. As in other diseases, certain miRNAs can participate in different pathophysiological events in more than one organ, and therefore, it is not uncommon that IPA analysis reveals additional diseases besides liver disease that might be associated with certain miRNAs. Additional enrichment analysis was completed to further characterize the relationship between dysregulated miRNAs in ZZ plasma derived EVs and HSCs activation. The results of network analysis are presented in Fig. 3e. Consistent with our hypothesis, the IPA analysis revealed molecular networks targeted by miRNAs involved in HSCs activation. The networks contained genes predicted to be involved in ECM synthesis, Inflammatory pathways and cellular growth and proliferation.

**Table 2:** Significantly dysregulated miRNAs in ZZ plasma-derived EVs

Up-regulated	Fold change	Down-regulated	Fold change
hsa-miR-125a-5p.hsa-mir-125a	5.36697164	hsa-miR-658.hsa-mir-658	−5.9859744
hsa-miR-125b-5p.hsa-mir-125b-1	5.18329317	hsa-miR-4488.hsa-mir-4488	−3.378354
hsa-miR-125b-5p.hsa-mir-125b-2	5.18340772	hsa-miR-4800-5p.hsa-mir-4800	−3.8837823
hsa-miR-139-5p.hsa-mir-139	3.30934504	hsa-miR-324-5p.hsa-mir-324	−3.1512944
hsa-miR-151a-5p.hsa-mir-151a	3.45601808	hsa-miR-3656.hsa-mir-3656	−2.9581834
hsa-miR-151b.hsa-mir-151b	3.61474378	hsa-miR-6809-5p.hsa-mir-6809	−6.8978544
hsa-miR-30c-5p.hsa-mir-30c-1	2.59281873	hsa-miR-4516.hsa-mir-4516	−3.2291639
hsa-miR-30c-5p.hsa-mir-30c-2	2.59370236	hsa-miR-218-5p.hsa-mir-218-1	−3.3050034
hsa-miR-335-3p.hsa-mir-335	5.55148985	hsa-miR-218-5p.hsa-mir-218-2	−3.3052121
hsa-miR-339-5p.hsa-mir-339	5.07518785	hsa-miR-6867-5p.hsa-mir-6867	−3.8457938
hsa-miR-340-3p.hsa-mir-340	3.22016136	hsa-miR-451a.hsa-mir-451a	−2.6098699
hsa-miR-128-3p.hsa-mir-128-1	2.5067686	hsa-miR-6510-5p.hsa-mir-6510	−6.6429613
hsa-miR-328-3p.hsa-mir-328	4.1125644	hsa-miR-96-5p.hsa-mir-96	−2.506894
hsa-miR-99a-5p.hsa-mir-99a	2.78701394	hsa-miR-936.hsa-mir-936	−6.7286907
hsa-miR-191-3p.hsa-mir-191	3.57966715	hsa-miR-16-5p.hsa-mir-16-2	− 1.9759375
hsa-miR-128-3p.hsa-mir-128-2	2.29874622	hsa-miR-4793-5p.hsa-mir-4793	−4.5613285
hsa-miR-4433b-5p.hsa-mir-4433b	5.40370557	hsa-miR-4298.hsa-mir-4298	−4.1782314
hsa-miR-4446-3p.hsa-mir-4446	3.07252979	hsa-miR-16-5p.hsa-mir-16-1	−1.9774818
hsa-miR-130b-5p.hsa-mir-130b	7.72943936	hsa-miR-4306.hsa-mir-4306	−2.9663986
hsa-miR-222-3p.hsa-mir-222	2.17946999	hsa-miR-15b-5p.hsa-mir-15b	−2.1452906
hsa-miR-26b-3p.hsa-mir-26b	4.44725049	hsa-miR-106b-5p.hsa-mir-106b	−2.0233933
hsa-miR-150-5p.hsa-mir-150	3.4166121		
hsa-miR-3615.hsa-mir-3615	2.17274498		

Positive and negative values for Log_2_(FC) represent miRNAs up-regulated or down-regulated, respectively, in ZZ individuals

### Plasma derived-EVs from AATD individuals activate hepatic stellate cells in vitro

**Fig. 4:**
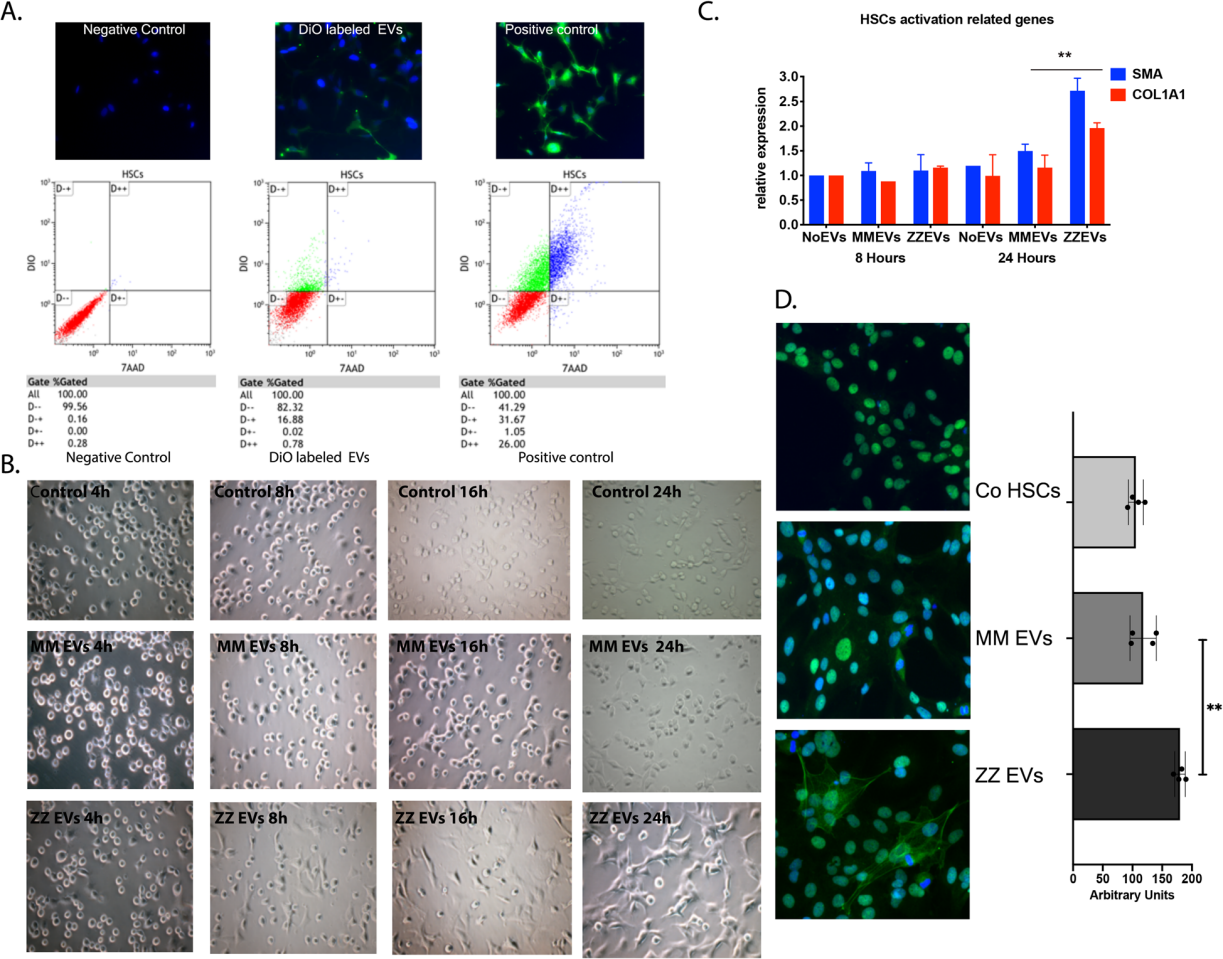
Plasma derived-EVs from AATD individuals activate hepatic stellate cells in vitro. **a** Fluorescent microscopic images demonstrating interaction of DiO-labeled EVs with recipient HSCs. Images were taken 1 h after EVs were added to the culture medium. Images were captured 20× objective. Flow cytometric analysis confirmed a population of green fluorescence positive cells present in EV-fed groups. **b** Representative phase contrast images of morphology of quiescent stellate cells incubated with PBS (top row), c (middle row) and ZZ plasma derived-EVs (bottom row) during 24 h of incubation. **c** LX-2 cells were incubated with and without isolated MM and ZZ plasma derived-EVs and the expression of α-SMA and Col1a1 were measured using real-time PCR after 24 h of incubation. **p = 0.0041 **d** Immunofluorescence images of the expression levels of *α*-SMA in LX-2 cells incubated with and without isolated MM and ZZ plasma derived-EVs followed by *α*-SMA fluorescent intensity. Green fluorescence indicates LX-2 cells that express α-SMA and blue spots indicate nuclei stained with DAPI (Images were captured 40× objective). **p = 0.0020

In order to evaluate the interaction and possible uptake of plasma derived-EVs by cultured HSCs, purified EVs were labeled with DiO and incubated with the LX-2 cells [26] for fluorescence microscopy and flow cytometry analysis. Fluorescence microscopy images of the treated LX-2 cells exhibited green spots or small patches. In contrast, the HSCs treated with DiO alone showed a homogeneous green fluorescent staining. HSCs incubated with DiO-labeled EVs were also analyzed by flow cytometry to provide quantitative measurement of the above internalization. HSCs treated with DiO-labeled EVs produced lower fluorescent intensity compared to cells stained with DiO only (Fig. 4a). Residual dye was substantially eliminated after a second wash, suggesting that the signal from DiO EVs was from the DiO associated with EVs. To further evaluate the bioactivity of the plasma-derived EVs, quiescent LX-2 cells and iPSc-derived HSCs were incubated with or without MM or ZZ plasma-derived EVs for 24 h and cell morphology was monitored by light microscopy. Images taken from different treatment conditions showed that quiescent iPSc-derived HSCs incubated with ZZ plasma-derived EVs exhibited an elongated, fibroblast-like shape after 8 h, indicating an activated state. In contrast, the iPSc-derived HSCs incubated without EVs or with MM plasma-derived EVs exhibited a more rounded morphology suggestive of a more quiescent state (Fig. 4b). The activation status of HSCs was determined by quantifying α-SMA and Col1A1 mRNA expression, markers of stellate cell activation [27]. They were found to be significantly increased by incubation with ZZ plasma-derived EVs after 24 h compared to control and MM plasma-derived EV treatment (Fig. 4c). Next, we investigated the effect of ZZ plasma-derived EVs on the protein levels of α-SMA in HSCs using fluorescence microscopy. Consistent with our findings above, only cells incubated with ZZ plasma-derived EVs had significant increase of α-SMA green fluorescent signal compared to controls (Fig. 4d).

### NF-κB and JAK/STAT-dependent regulation of activation and migration of HSCs

**Fig. 5:**
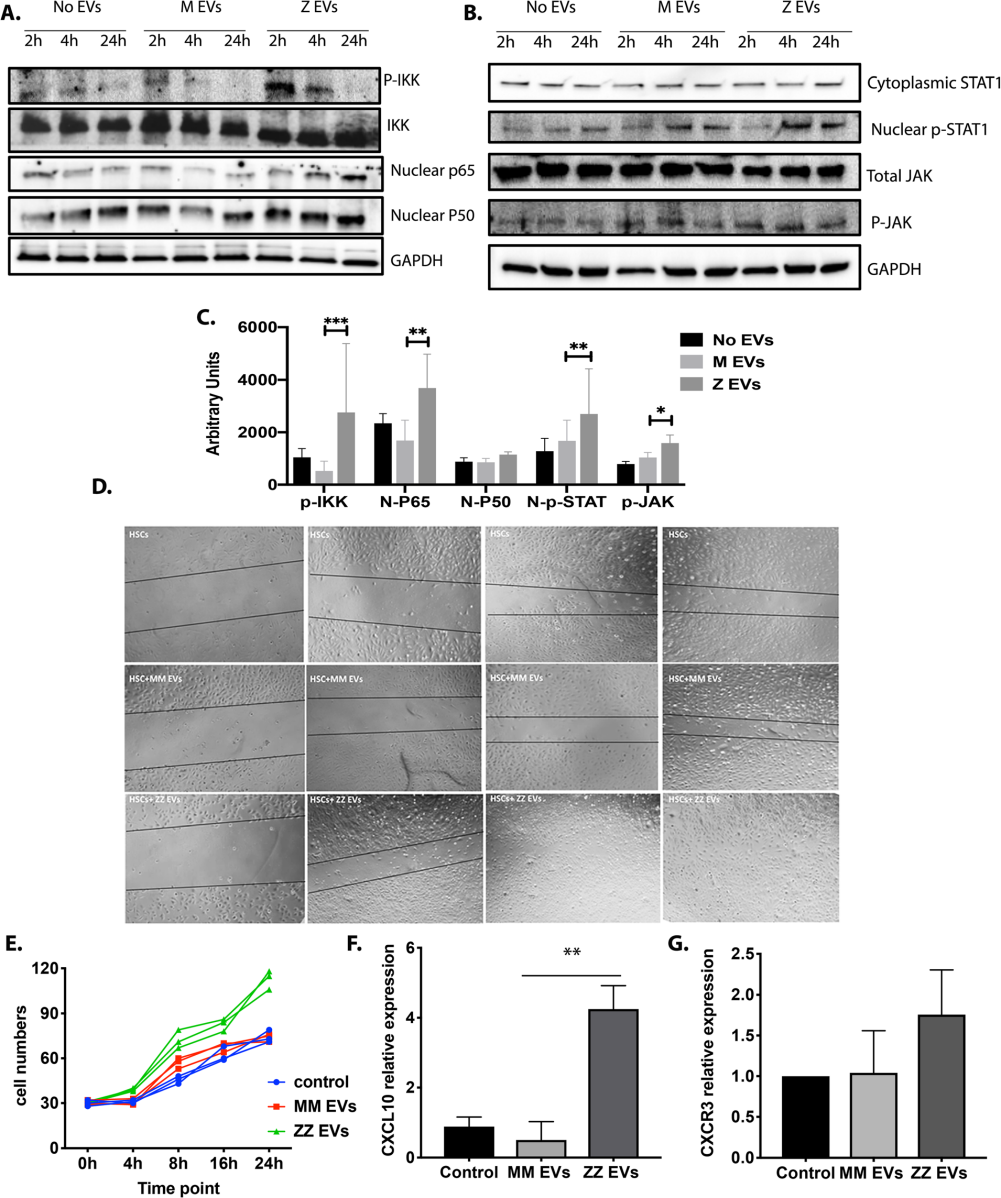
NF-κB and JAK/STAT-dependent regulation of activation and migration of HSCs. (**a and b**) The iPSCs derived-HSCs were incubated with and without isolated MM and ZZ plasma derived-EVs and the protein expression of the phospho-IKK, total IKK, nuclear p65, nuclear p50, cytoplasmic STAT1 and JNK, and nuclear phospho-STAT-1 and phosphor-JNK were determined during 24 h of incubation by Western blot analysis. GAPDH protein level was used as loading control. The assays were performed at least three times with similar results. **c** Blot quantification bar graphs. **d** Wound-healing and **e** cell proliferation assays of HSCs treated with or without EVs derived from MM and ZZ plasma samples or PBS control during 24 h of incubation. **f and g** LX-2 cells were incubated with and without isolated MM and ZZ plasma derived-EVs and the expression of CXCL10 and CXCR3 were measured using real-time PCR after 24 h of incubation, *p < 0.05, **p < 0.005, ***p < 0.0005

Although LX-2 cell line enjoys great popularity among researchers interested in the elucidation of mechanisms underlying stellate cell biology and liver fibrosis, it expresses a-SMA, vimentin, GFAP, and PDGFRb suggesting that cell line retains key features of activated/transdifferentiated HSC. As a consequence, key results obtained with these cell line should be validated in primary cells if possible [26]. Therefore, to investigate the signaling pathways likely to be responsible for HSCs activation by ZZ plasma-derived EVs, we quantified the signaling molecules involved in the canonical NF-κB and JAK/STAT signaling pathways in iPSC-derived HSCs treated with or without plasma-derived EVs. At different timepoints following incubation with no EVs, MM plasma derived EVs or ZZ plasma derived EVs, HSCs were subjected to western blot analysis and intracellular levels of phosphorylated of IKK, which is central to the NF-κB signaling pathway, and nuclear translocation of p50 and p65 subunits [28], were measured. Phosphorylation of IKK and nuclear translocation of p50 and p65 subunits were all significantly increased in iPSC-derived HSCs treated with ZZ plasma-derived EVs compared to HSCs treated with MM plasma-derived EVs and control HSCs (Fig. 5a and c). The phosphorylation of JAK and nuclear STAT1 were also slightly elevated in iPSC-derived HSCs treated with ZZ plasma-derived EVs compared to control groups (Fig. 5b and c). Thus, ZZ plasma-derived EVs regulate the transition process of HSCs from a quiescent to activated state through canonical NF-κB and JAK/STAT signaling pathways. Wound healing assays and cell counting revealed that ZZ plasma-derived EVs significantly enhanced the migratory and contractile capacities of LX2 cells (Fig. 5d and e). Furthermore, HSCs migration and proliferation require stimulation of the CXCR3 receptor on HSCs with the CXCL10 ligand [29]. Therefore, we detected the over expression of CXCL10 mRNA, which is downstream of the NF-κB signaling pathway, to confirm migratory augmentation of HSCs after 24 h of incubation with ZZ plasma-derived EVs by qPCR (Fig. 5f). We have also confirmed the expression of CXCR3 receptor in our HSC model (Fig. 5g).

### Plasma CXCL10 and EV-associated cytokine levels are associated with AATD-mediated liver fibrosis in AATD individuals

**Fig. 6:**
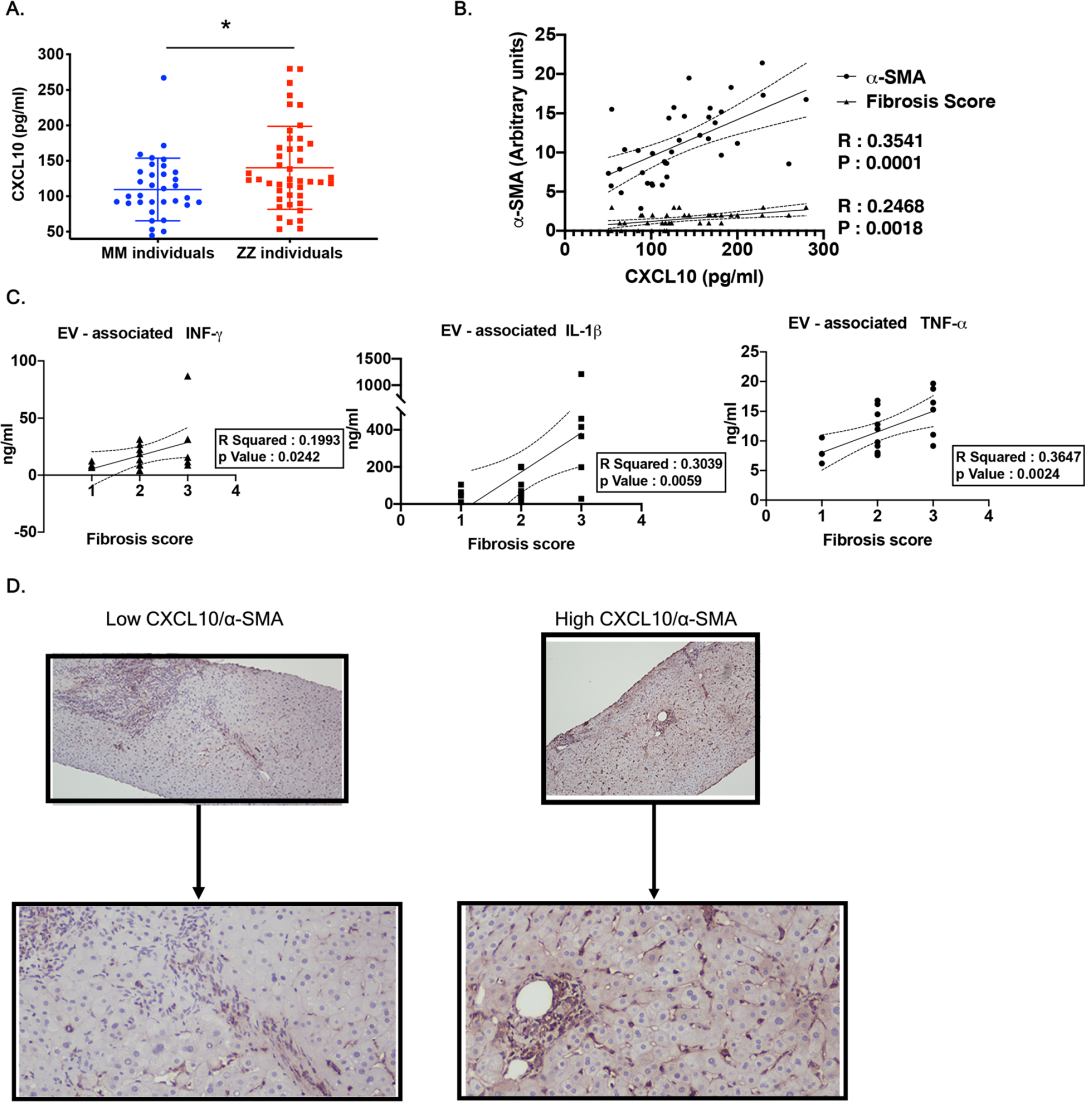
High CXCL10 plasma levels is associated with AATD mediated-liver fibrosis in AATD individuals. **a** Dot plot graph illustrating differences in the serum CXCL10 levels of ZZ individuals compared to MM healthy controls, *P = 0.0131. **b** The correlation between serum CXCL10 levels and *α*-SMA immunoreactivity on the liver biopsy from ZZ individuals. **c** The correlation between EV associated INF- *γ*, IL1- *β* and TNF- *α* levels of and liver fibrosis score of ZZ individuals. **d** Mild and severe perisinusoidal *α*-SMA staining on the liver biopsy from ZZ individuals with low and high CXCL10 plasma levels respectively

It has been shown that the IFN- *γ* inducible chemokines CXCL9, CXCL10, and CXCL11 are up-regulated in patients with chronic liver diseases, and their serum levels are closely associated with the degree of hepatic fibrosis. Among these chemokines which share the receptor CXCR3, CXCL10 appears to be profibrogenic by direct effects on HSCs. Within the liver, hepatic stellate cells express CXCR3 which promotes HSC migration through interaction with CXCL10 ligand [29]. To further identify the potential association between CXCR3-associated CXCL10 and HSC activation and migration, we evaluated the plasma levels of CXCL10 in samples obtained from 44 ZZ individuals and 36 healthy MM controls. We observed that the mean CXCL10 plasma level was significantly higher among the ZZ individuals compared to the healthy MM group (Fig. 6a). Among the ZZ individuals, we quantified the association between activated HSCs by investigating intrahepatic *α*-SMA positive cells as well as fibrosis score and the plasma levels of CXCL10 in ZZ individuals from whom biopsy samples were available. As expected, we observed that the numbers of intrahepatic *α*-SMA positive cells and fibrosis score are corelated to the plasma levels of CXCL10 (Fig. 6b). We also identified correlation between plasma EV- associated upregulated cytokines and fibrosis score among the ZZ individuals (Fig. 6c). Immunohistochemical staining of the liver biopsies from ZZ individuals demonstrated that those with higher plasma levels of CXCL10 had substantially greater intrahepatic *α*-SMA positive cells compared to those with lower plasma levels (Fig. 6d).

## Discussion

In this study 1) Plasma circulating EVs from ZZ AATD individuals have pathologic immunomodulator contents; 2) Pro-fibrotic cytokines and microRNAs are upregulated in ZZ plasma-derived EVs; 3) ZZ plasma-derived EVs contribute to HSCs activation and liver inflammation, thus AATD-mediated liver fibrosis pathology.

Recent studies have shown that in addition to AATD-mediated liver disease and lung disease, immune complications and systemic inflammation are also prevalent in many patients with AATD [30]. It is thought that increased production of proinflammatory cytokines and immunomodulators released by injured lung and liver parenchymal cells, is involved in inflammatory liver diseases physiopathology [31]. Although these secreted proinflammatory factors play critical roles in intercellular communications, here we focus on the contribution of EVs as unappreciated cell-cell communication mediators to AATD related liver disease.

In this study, we used serial ultracentrifugation and filtration to isolate EVs from plasma, which is a state-of-the-art method that does not modify the original sample. We used a variety of EV characterization methods based on morphology, size, number, and expression of EV-enriched proteins. Electron microscopy and NTA analysis showed that EVs isolated from plasma samples are < 200 nm in size. This particle size is consistent with previously reported exosome subpopulation size measurements and is in close agreement with EM data. Western blot analysis revealed that among the surface molecules on plasma derived-EVs, Tumor Susceptibility Gene 101 (TSG101) was enriched in the EV population isolated from the plasma sample of ZZ individuals compared to controls. Endosomal Sorting Complexes Required for Transport (ESCRT), such as TSG101 recognize and sort ubiquitinated cargo destined for lysosomal degradation. The increased levels of TSG101 expression in the EVs from ZZ individuals could be due to the presence of ZAAT ubiquitinated cargo in ZAAT expressing cells as we have previously shown [32].

A large body of evidence has supported the involvement of EVs in the protein aggregate spreading, contributing to the disease progression [33]. We observed that AATD individuals exhibited a significant amount of AAT aggregates in plasma derived EVs providing evidence of their pathogenic potential. AAT is primarily produced by hepatocytes. Presence of AAT aggregates in plasma EVs shows that injured hepatocytes are one of the major sources of plasma EVs in AATD individuals. In our study and consistent with previous studies [34], elevated levels of CRP indicate a systemic inflammatory state in AATD individuals compared to control. Elevated cytokine levels are one of the characteristics of chronic immune system activation [35]. AAT is a known acute-phase protein with important anti-inflammatory and immune-modulating properties. Hence, considering the immune-modulating function of AAT an immune activation plays a role in the inflammation of different tissues especially lungs in individuals with AATD [36]. While cytokine levels in the plasma of AATD individuals have been the subject of investigation, the results presented here demonstrate that a significant number of cytokines/chemokines are not released in free form but are associated with EVs. Given the increased stability of molecules carried by enclosed membrane compartments such as EVs [37], it is plausible that association of cytokines and chemokines with EVs would imply their increased half-life, as well as their wider distribution to specific target cells and organs distal from the producer cells. The higher concentration of EV associated INF- *γ*, IL1- *β*, TNF- *α* and IL-8 in the plasma of AATD individuals indicates a potentially significant role of EVs in mediating the effect of these cytokines on recipient cells. These data also indicate that activated immune cells from different tissues such as lungs can be the other source of plasma EVs in AATD individuals. These cytokines are initiators of acute phase inflammatory response in the liver, including apoptosis of hepatocytes, steatosis and inflammation as well as activation of HSCs [38]. Our result show that the plasma levels of EV-associated proinflammatory cytokines such as INF- *γ*, IL1- *β*, TNF- *α* are correlated with the fibrosis levels of the liver in AATD individuals. Thus, it demonstrates that EVs associated proinflammatory cytokines correlate with progression of liver inflammation in AATD individuals.

MicroRNAs (miRNA) are short, non-coding, RNA that function as regulatory molecules and are involved in a series of vital processes and in the pathogenesis of various human diseases. Previous studies have demonstrated the significant role of miRNA in liver diseases [39]. It has been also shown that there is a correlation between the expression of inflammatory miRNAs in the peripheral blood mononuclear cells and severity of lung disease in AATD individuals [40]. In this study plasma circulating EV-associated miRNAs were profiled in healthy MM individuals compared to ZZ individuals with liver disease. The expression pattern of plasma circulating EV-associated miRNAs is substantially different in AATD mediated liver disease. These miRNAs are consistent with known characteristics of ZZ individuals with liver disease such as HSCs activation, fibrosis and cirrhosis, and HCC which shows the possibility of using EVs as a potential source of biomarkers for early diagnosis of disease progression. Importantly, we identified that of the 44 miRNAs whose levels change in the plasma EVs of AATD individuals, 5 which were higher in AATD individuals (miR-125a, miR-125b1, miR-125b2, miR-128 and miR-130b) and 1 lower (miR-6809) appear to play important roles in liver fibrosis [41–45]. A large body of evidence supports the implication of the miR-125 family and miR-130b [46] in different liver diseases, including ALF [47], non-alcoholic fatty liver disease [48], cholestasis [49], and HCC [50]. miR-125b has been also shown to have a central role in the regulation of HSC’s trans-differentiation during liver fibrogenesis and a promotive function in HSC activation and production of *α*-SMA during the progression of liver fibrosis [41]. Furthermore, increased expression of miR-128 has also been observed during liver fibrosis and found to be associated with chemokines and chemokine receptors regulating inflammation during fibrosis [51]. Other miRNAs are concomitant with liver homeostasis, including miR-6809-5p that has been shown to play a role in suppressing HCC cell growth. miR-6809-5p directly targets the FLOT1 gene through inhibiting signaling pathways including JNK and NF-κB/p65 [44]. Our IPA analysis results consistent with our hypothesis, also revealed NF-κB molecular network is being targeted by a group of differentially expressed miRNAs (Supplementary Figure 2).

Downregulation of flotillin protein in human monocytes and fibroblasts also impairs collagen uptake by promoting lysosomal degradation of the endocytic collagen receptors, a characteristic of many fibrotic diseases like liver fibrosis [52]. Such an alteration in circulating miRNA profile could be used to evaluate the risk of developing liver disease in AATD individuals, which may improve prediction and prevention among individuals at high risk of the disease.

In liver fibrosis, altered cellular cross-talk is a key feature of pathological wound healing and fibroblast activation and proliferation [53]. In this study, we describe that activated immune cells resulting from AATD-mediated immune complications and injured hepatocytes are a source of pro-inflammatory EVs and demonstrate paracrine effects of EVs on HSCs activation and trans-differentiation. Similarly, colangiocyte-derived EVs were found to promote HSC activation and cholestatic liver fibrosis [54]. HSCs are pericyte-like cells in the space of Dissé (located between hepatic sinusoidal endothelial cells and hepatocytes), and they are usually in the quiescent state. However, HSCs which undergo trans-differentiation and activation are characterized by increased migratory capacity and deposition of extracellular matrix components during liver inflammation [55]. Extracellular signals from injured resident epithelial cells and inflammatory cells further play an important role in modulating HSC activation [56]. EVs are increasingly implicated in a variety of liver pathobiological conditions, including liver cirrhosis, portal hypertension, and HCC [57]. Our study adds to these existing models by showing a role for plasma circulating EVs in signaling relevant to AATD-mediated liver fibrosis through mechanistic studies that elucidate possible signaling pathways involved in EV-mediated HSC activation.

Many immunomodulators, including TNFα and IL-1β, promote activation of HSCs by stimulating nuclear factor-κB (NF-κB) and downregulating pro-apoptotic genes [58–61]. NF-κB represents a family of inducible transcription factors, which regulates a large group of genes involved in different processes of the immune and inflammatory responses. The primary mechanism for canonical NF-κB activation is nuclear translocation of the NF-κB executor transcription factors. This process subsequently activates the transcription of many pro-inflammatory genes [28]. Here, our western blot analysis revealed the nuclear translocation of p50 and p65 transcription factors in HSCs treated with ZZ plasma-derived EVs. Furthermore, similar results were obtained by examining the JAK/STAT signaling pathway in HSCs treated with ZZ plasma-derived EVs compared to no treatment or treatment with healthy MM plasma-derived EVs. We found that ZZ plasma-derived EVs elevated the nuclear levels of phosphorylated STAT-1 and JNK, indicating activation of JAK/STAT pathway. The perpetuation phase of HSC activation overlaps with the signals of survival, migration, proliferation, fibrogenesis, and retention of an activated phenotype as the injury stimulus persists and activates critical pathways of HSC proliferation/migration such as the JAK/STAT signaling pathway [59].

Cell migration is a crucial step in the development and aggravation of several diseases, from organ fibrosis to cancer. Persistent HSCs migration contributes to the progression of fibrotic areas in the liver, resulting in excessive tissue remodeling and fibrotic scarring [62]. Therefore, we evaluate the migration of HSCs treated with or without ZZ plasma-derived EVs compared to healthy MM plasma-derived EVs. Our migration study based on a wound healing assay revealed that HSC’s migratory function and proliferation rate are augmented by incubation with ZZ plasma-derived EVs compared to control. Interestingly, quantitative PCR analysis showed upregulation of CXCL10 in the activated HSCs, consistent with our ELISA results showing elevated plasma levels of CXCL10 in AATD individuals compared to control population. Previous studies have shown that CXCL10 stimulation of the CXCR3 receptor leads to migration of primary HSCs [29, 63]. It has also been reported that IFN- *γ* induces CXCL10 expression through activation of JAK/STAT-1a pathway [64, 65], which is also activated in HSCs treated with ZZ plasma-derived EVs in our study. Thus, IFN- *γ* associated with EVs from ZZ plasma-derived EVs may contribute to induction of CXCL10 production by activated HSCs, regulating their migratory function in an autocrine effect. In response to their activation HSCs show an intense cytoplasmic *α*-SMA immunoreactivity. *α*-SMA is a specific marker for activated HSCs that show a myofibroblastic phenotype [66]. *α*-SMA-positive HSCs are responsible for fibrotic tissue accumulation in human chronic liver disease and correlate with the extent of fibrosis [67]. Our data agree with the models of fibrogenesis proposed by several authors where the activation of HSCs is the central event in hepatic fibrosis and shows that the number of *α*-SMA-positive HSCs mildly correlates with plasma levels of CXCL10 in AATD individuals enrolled in our study. On the other hand, our data suggests that during phases of hepatic insult, activated HSCs may amplify the response to liver injury producing pro-inflammatory cytokines such as CXCL10.

While various imaging modalities are widely used for assessment of liver fibrosis, these techniques lack sensitivity and specificity for early stages of fibrosis and are not able to determine liver inflammation and hepatocellular injury [68]. Studies have shown that sampling errors alone causes a diagnostic error greater than one stage of fibrosis in as high as in 33.1% of the biopsy samples from liver patients [69]. Furthermore, given the heterogeneity of liver fibrosis, a biopsy may not reflect the entire liver and therefore individual biopsies often missed fibrotic areas present elsewhere. Processing of tissue samples, including fixation and paraffin embedding, may also limit subsequent molecular analysis. Finally, tissue biopsy limits tissue analysis to a single point in time, whereas a time series measurement of fibrosis of the same individual to monitor fibrosis progression is helpful to track the potential response to the treatment [68]. Therefore, now is the time to develop innovative ways that may perhaps replace biopsy as an invasive gold standard for comparisons, and come up with more precise and noninvasive novel metrics to evaluate fibrosis levels. For example, Liquid biopsy, referring to analysis of patient liquid specimens such as blood instead of surgical biopsy specimens, offers the promise of diagnosis in a less invasive manner. With the progress in the novel therapies for various chronic liver diseases, the development of liquid biopsy tools has evolved into a key priority in the field of hepatology such as AATD-mediated liver disease. Non-invasive reliable biomarkers that can supplement and eventually replace invasive liver biopsy are crucial for selection of the high risk AATD patient and monitoring their liver condition. Circulating EVs have emerged as attractive liquid biopsy candidates because their cargo contains many of the key characteristics of an ideal biomarker [68]. In this study we successfully isolated and purified EVs from AATD plasma with a ZZ genotype and healthy MM individuals, which were characterized by various approaches to prove the high quality of the isolated EVs.

## Conclusion

**Fig. 7:**
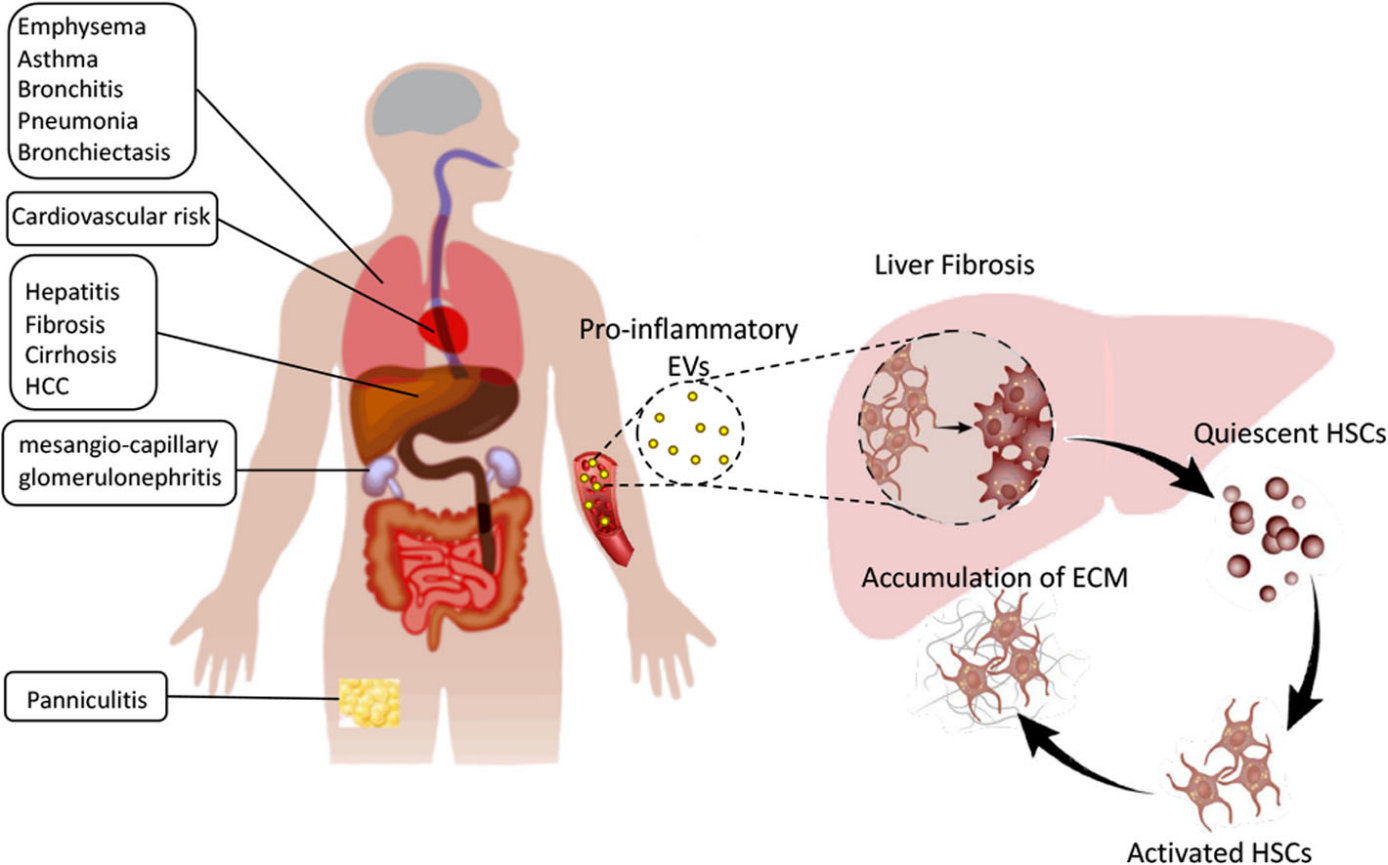
Plasma derived EV mediated liver fibrosis in AATD individuals. EVs are small vesicular structures that are shed by different cells and provide various autocrine and paracrine signaling cues. AATD mediated immune complications, such as lung disease, panniculitis, cardiovascular risks, and mesangial-capillary glomerulonephritis suggest a contribution from a variety of activated immune cells as well as parenchymal cells. EVs released by activated immune cells and injured parenchymal cells carry a pro-fibrotic cargo with the ability to promote liver fibrosis through activation of HSCs. AATD plasma derived EVs induced HSC trans differentiation and activation leading to deposition of ECM and fibrotic phenotype

Our study presents evidence for a new role of plasma circulating EVs in modulating the immune response of the liver through HSC activation (Fig. 7). Therefore, plasma circulating EVs may represent a target for anti-inflammatory therapy in liver fibrosis. This is related to the fact that plasma-derived EVs contain dysregulated immunomodulator contents, such as cytokines and miRNAs, in AATD individuals. This later finding is important since there are very few human studies in the literature that evaluated biomolecular cargo of plasma EVs as biomarkers of liver disease. We are aware of no studies evaluating plasma-derived EV cargo for biomarkers of AATD-mediated liver disease. Further studies should be conducted to validate some candidate prognostic markers in a separate cohort of AATD individuals and confirm the functional roles of EVs in the AATD-mediated liver disease. More patients should be included to reduce bias and enhance the reproducibility and reliability of the study, particularly for proteomic analysis. Limited sample size may compromise the robustness.

## Supplementary information

**Additional file 1: Figure Supplementary 1.** Characterization of iPSC-derived HSCs. (A). Representative pictures showing iPSC-HSCs differentiation steps at different days. Scale bars, 200 μm. (B) Schematic representation of the differentiation protocol from day 0 to day 12; sequential stimulation with different growth factors. (C) HSCs related gene expression in Huh7, LX2, MM and ZZ individuals iPSCs-derived HSCs.

**Additional file 2: Figure Supplementary 2.** Molecular networks linking highly differentially expressed microRNAs (miRNAs) and their target genes involved in the regulation of NF-κB pathway between AATD individuals and healthy controls. Upregulated miRNAs are in red and downregulated miRNAs are green.

## Data Availability

All data generated or analyzed during this study are included in this published article [and its supplementary information files].

## Abbreviations

AAT: Alpha-1 antitrypsin
AATD: Alpha-1 antitrypsin deficiency
α-SMA: Alpha smooth muscle actin
CoL1A1: Collagen type 1 alpha 1
CPM: Counts per million
CRP: C-Reactive Protein
DMEM: Dulbecco’s Modified Eagle’s Medium
ER: Endoplasmic reticulum
ES: Effective size
ESCRT: Endosomal sorting complexes required for transport
EV: Extracellular vesicle
HSC: Hepatic stellate cell
IPA: Ingenuity Pathway Analysis
iPSc: Induced pluripotent stem cell
LRAT: Lecithin: Retinol acyl transferase
miRNA: MicroRNAs
NTA: Nanoparticle tracking analysis
PAS + D: Periodic acid-Schiff diastase resistant
SD: Standard deviation
TEM: Transmission electron microscopy
TIMP: Tissue inhibitor of metallopeptidase inhibitor 1
TSG101: Tumor susceptibility gene 101
ZAAT: Z-variant alpha-1 antitrypsin
