# Supplementary figures and images for: Alpha-1 antitrypsin deficient individuals have circulating extracellular vesicles with profibrogenic cargo
Source: Cell Commun Signal. 2020 Sep 4;18:140. doi: 10.1186/s12964-020-00648-0 (PMC7487708; doi:10.1186/s12964-020-00648-0)

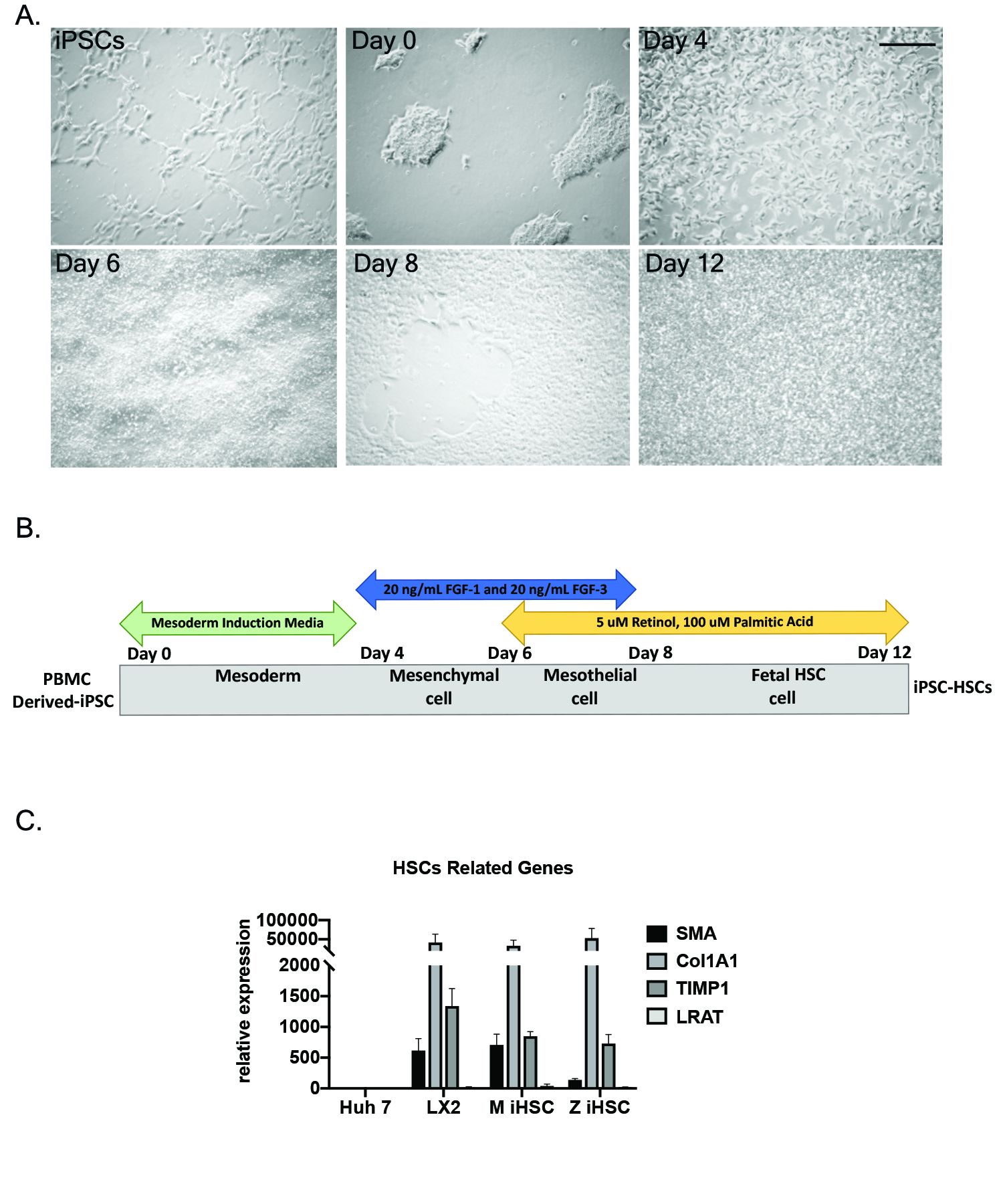

Supplement: Supplementary file 2 — Additional file 1: Figure Supplementary 1. Characterization of iPSC-derived HSCs. (A). Representative pictures showing iPSC-HSCs differentiation steps at different days. Scale bars, 200 μm. (B) Schematic representation of the differentiation protocol from day 0 to day 12; sequential stimulation with different growth factors. (C) HSCs related gene expression in Huh7, LX2, MM and ZZ individuals iPSCs-derived HSCs. [file 12964_2020_648_MOESM2_ESM.tif]

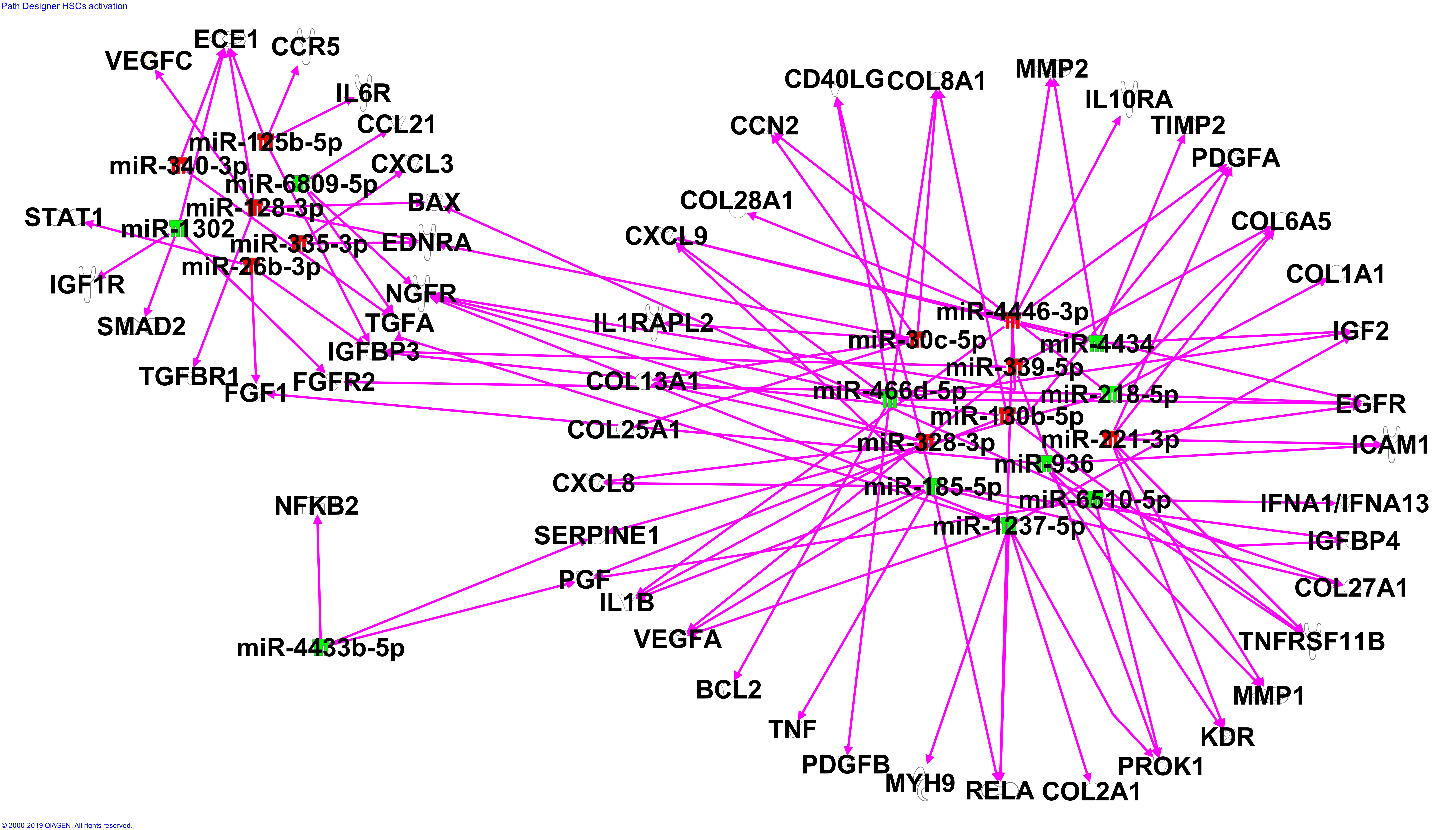

Supplement: Supplementary file 3 — Additional file 2: Figure Supplementary 2. Molecular networks linking highly differentially expressed microRNAs (miRNAs) and their target genes involved in the regulation of NF-κB pathway between AATD individuals and healthy controls. Upregulated miRNAs are in red and downregulated miRNAs are green. [file 12964_2020_648_MOESM3_ESM.tif]
